# The Effect of Tenofovir on Vitamin D Metabolism in HIV-Infected Adults Is Dependent on Sex and Ethnicity

**DOI:** 10.1371/journal.pone.0044845

**Published:** 2012-09-12

**Authors:** Karen Klassen, Adrian R. Martineau, Robert J. Wilkinson, Graham Cooke, Alan P. Courtney, Mary Hickson

**Affiliations:** 1 Institute of Health and Biomedical Innovation, Queensland University of Technology, Brisbane, Australia; 2 Imperial College Healthcare NHS Trust, London, United Kingdom; 3 Department of Medicine, Imperial College London, United Kingdom; 4 Barts and The London Medical School of Medicine and Dentistry, Queen Mary, University of London, London, United Kingdom; 5 MRC National Institute for Medical Research, London, United Kingdom; University of Tennessee, United States of America

## Abstract

**Background:**

Tenofovir has been associated with renal phosphate wasting, reduced bone mineral density, and higher parathyroid hormone levels. The aim of this study was to carry out a detailed comparison of the effects of tenofovir versus non-tenofovir use on calcium, phosphate and, vitamin D, parathyroid hormone (PTH), and bone mineral density.

**Methods:**

A cohort study of 56 HIV-1 infected adults at a single centre in the UK on stable antiretroviral regimes comparing biochemical and bone mineral density parameters between patients receiving either tenofovir or another nucleoside reverse transcriptase inhibitor.

**Principal Findings:**

In the unadjusted analysis, there was no significant difference between the two groups in PTH levels (tenofovir mean 5.9 pmol/L, 95% confidence intervals 5.0 to 6.8, versus non-tenofovir; 5.9, 4.9 to 6.9; p = 0.98). Patients on tenofovir had significantly reduced urinary calcium excretion (median 3.01 mmol/24 hours) compared to non-tenofovir users (4.56; p<0.0001). Stratification of the analysis by age and ethnicity revealed that non-white men but not women, on tenofovir had higher PTH levels than non-white men not on tenofovir (mean difference 3.1 pmol/L, 95% CI 5.3 to 0.9; p = 0.007). Those patients with optimal 25-hydroxyvitamin D (>75 nmol/L) on tenofovir had higher 1,25-dihydroxyvitamin D [1,25(OH)_2_D] (median 48 pg/mL versus 31; p = 0.012), fractional excretion of phosphate (median 26.1%, versus 14.6; p = 0.025) and lower serum phosphate (median 0.79 mmol/L versus 1.02; p = 0.040) than those not taking tenofovir.

**Conclusions:**

The effects of tenofovir on PTH levels were modified by sex and ethnicity in this cohort. Vitamin D status also modified the effects of tenofovir on serum concentrations of 1,25(OH)_2_D and phosphate.

## Introduction

Whilst antiretroviral therapy (ART) has transformed the prognosis of HIV-1 infection, metabolic consequences such as osteoporosis, of both infection and its therapy, have become more common. A meta-analysis found that the prevalence of osteoporosis in HIV-infected subjects was over three times higher than in HIV-uninfected persons. [1] The exact mechanisms are unclear, but a combination of factors are thought to contribute to decreased bone mineral density in HIV-infected people including lifestyle (e.g. diet, exercise, smoking and alcohol), genetic influences, as well as direct effects of HIV infection [2] and administration of antiretrovirals, such as Tenofovir disoproxil fumarate (TDF) [1], [3], [4], protease inhibitors (PIs) [1] and zidovudine. [5] Administration of TDF has also been associated with renal phosphate wasting, [6] osteomalacia [7], [8] and more recently, increased parathyroid hormone (PTH) levels [9]–[11]. The mechanism behind the potential alteration of calcium and phosphate homeostasis by TDF is not well understood. Additionally, it remains unclear whether renal phosphate wasting is related to either reduced bone mineral density and/or hyperparathyroidism in patients receiving TDF.

The aim of this study therefore was to examine the effect of tenofovir on PTH, calcium and phosphate homeostasis, vitamin D metabolism and bone mineral density by comparing patients receiving TDF with patients not receiving TDF. Furthermore, we aimed to investigate whether these effects are modified by ethnicity, age and sex.

## Methods

### Design

We conducted a prospective cohort study.

### Participants

Adults (aged >18 years) with HIV-1 infection attending a large UK outpatient clinic. Patients were recruited between February and September 2010 if they had received a non-nucleoside reverse transcriptase inhibitor (NNRTI)-based antiretroviral regimen (either with TDF or a nucleoside reverse transcriptase inhibitor (NRTI)) for greater than 12 months. Patients were excluded if they had significant renal, hepatic or thyroid dysfunction; concurrent major systemic illness (including malignancy and granulomatous infections); metabolic bone disease; were prescribed protease inhibitors, or were taking vitamin D supplements >400 IU/day during the preceding 4 months.

### Investigations Taken

Patients who gave written informed consent to participate were given a 24 hour urine collection bottle, had blood drawn, had weight and height measured using standardised techniques for calculation of body mass index (BMI), and completed questionnaires to determine exercise habits, dietary calcium intake [12], dietary vitamin D, demographics, sun exposure [13] and other lifestyle factors. Reference Nutrient Intakes (RNI) for calcium are defined as 700 mg/day for both adult men and women [14], therefore patients’ calcium intakes were categorized into “Meeting the RNI” or “Not Meeting the RNI”. Body composition and bone mineral density (BMD) of the lumbar spine and femurs were measured using a Lunar Prodigy dual energy x-ray absorptiometer (DXA) scanner (GE Healthcare, Madison, WI). The season in which the blood sample was obtained was classified as summer (June through August) and ‘other’ for all other months [13].

### Laboratory Methods

Serum 25-hydroxyvitamin D [25(OH)D] concentration (reference range; <50 nmol/L indicates deficiency; 50–75 nmol/L insufficiency; >75 nmol/L optimal) was determined by liquid chromatography-tandem mass spectrometry (LC-MS/MS [15]). Serum 1,25-dihydroxyvitamin D concentration (normal range, 20–50 pg/mL) was determined by radioimmunoassay following separation by high performance liquid chromatography (HPLC) as described in detail elsewhere [16]. Serum parathyroid hormone (PTH) concentration was determined by automated immunoassay on the Abbott Architect clinical chemistry analyser (*in vitro* chemiluminescent microparticle immunoassay, normal range, 1.1–6.8 pmol/L); bone specific alkaline phosphatase (BAP) concentration was determined by an enzyme-linked immunosorbent assay (ELISA) (Alkphase -B™; Quidel) (normal range, 15–41 u/L for males; 11–31 u/L for females). C-terminal Fibroblast growth factor-23 (FGF-23) concentration was also determined by a 2^nd^ generation ELISA (Immunotopics, San Clemente, CA, USA) (normal range <100 RU/mL) (analysed at the Royal Liverpool Hospital).

Urine measurements were determined for calcium (normal range, 2.50–7.50 mmol/24 hours), phosphate, urea and creatinine by automated Abbott Architect clinical chemistry analyser, and type 1 collagen N-terminal telopeptide Xlinks (NTX) was assayed by a competitive-inhibition ELISA (Osteomark™; Ostex International) (normal range, 1–51 BCE nmol/mmolCreatinine for males; 5–65 for pre-menopausal women; 5–131 post-menopausal women). The fractional excretion of phosphate and calcium were calculated (urine phosphate or calcium × serum creatinine/serum phosphate or calcium × urine creatinine) *100 for those patients who provided a urine and serum sample on the same day.

### Ethics

The study received ethical approval from the Charing Cross Research Ethics Committee (REC reference number: 09/H0711/89). All subjects provided written, informed consent.

### Statistical Methods

Chi Square, Fisher’s exact, Mann-Whitney and t-tests were used to assess between-group differences. We used single and multiple linear regression models for analysing predictors of PTH levels which have been shown in previous studies to influence PTH, including the following parameters: serum 25-hydroxyvitamin D concentration, season, ethnicity, sex, age, dietary calcium intake, TDF versus other NRTIs, NNRTI use (efavirenz (EFV) versus nevirapine (NVP)) and total body fat percentage (as determined by DXA). All variables with a p-value of <0.10 from the univariate analysis, in addition to age, NNRTI and TDF use, were entered into the multivariate model in a forward stepwise fashion using the Univariate General Linear Model. As the standard errors and confidence intervals for the model were all within an acceptable range, we ruled out multicollinearity. For ethnicity, two groups were considered: white and non-white due to smaller numbers of participants who identified as an ethnicity other than white. In the group of women, there were only non-white women, therefore the comparison of white vs. non-white was only possible within males.

The study aimed to recruit 56 participants, with 28 subjects in each study group. Assuming a significance level of 0.05, this was sufficient to detect a 2.5 pg/ml difference in PTH with a standard deviation of 3.3 [9]. All analyses were done using PASW Statistics 18 (SPSS, IBM, Somers, NY).

## Results

Twenty-eight patients receiving TDF and 28 receiving another NRTI (24 receiving abacavir (ABC), 3 receiving zidovudine (AZT) and 1 receiving didanosine (ddI) participated in this study. Table 1 describes the demographic and other HIV and ART-related characteristics of the cohort, and differences between the two groups. The two groups were well matched for age, sex, ethnicity and BMI, but more people on a non-TDF containing regime were taking NVP (85.7% of non-TDF group versus 50.0% of TDF group) and had biochemical analysis done during the summer months (85.2% of non-TDF group versus 35.7% of TDF group). In addition, patients had been receiving non-TDF containing regimes for longer (median 46 months) than those receiving TDF (median 35 months). Five patients in the NRTI group and 11 in the TDF group had osteoporosis or osteopenia of the lumbar spine or hips (p = 0.09), (see Table 1). Table 2 describes the biochemical characteristics and differences between the two groups. Patients receiving TDF were more likely to have lower overall fractional excretion of calcium (median 0.63%, IQR 0.44 to 1.01) than patients not taking TDF (1.21%, 0.96 to 1.38) (p = 0.004). No significant difference in PTH (pmol/L) levels was found between those taking TDF versus non-TDF (5.9, 95% Confidence Interval 4.9 to 6.9 versus 5.9, 95% CI 5.0 to 6.8; p = 0.98) nor in the proportion of patients with high PTH (25% versus 30%, p = 0.70) (see Table 2). Table S1 further describes the biochemical and medical characteristics of non-white males on TDF and other NRTIs.

**Table 1:** Demographic and HIV-related characteristics of the study cohort.

	Tenofovir	Other NRTIs	P-value
	N = 28	N = 28	
Male Sex, n (%)	23 (82.1)	20 (71.4)	0.34
Ethnicity: white, n (%)	18 (64.3)	16 (57.1)	0.76
Black, n (%)	7 (25.0)	8 (28.6)	
Other, n (%)	3 (10.7)	4 (14.3)	
White males, n	18	16	
Black males, n	3	1	
Other ethnicity males, n	2	3	
Age, years	46 (±9)	44 (±9)	0.50
Months since HIV diagnosis	130 (±81)	145 (±64)	0.45
CD4 count/mm^3^	583 (±186)	687 (±235)	0.07
eGFR (MDRD formula, mL/min/1.73 m^2^) (range)	92 (76–112)	94 (69–118)	0.58
Months on NRTI^†^	35 (24–57)	46 (42–68)	0.013*
Nevirapine, n (%)	14 (50.0)	24 (85.7)	0.004*
Efavirenz, n(%)	14 (50.0)	4 (14.3)	
Season (summer), n (%)	10 (35.7)	20 (71.4)	0.004*
Outside in daylight hours <30 min/day, n (%)	10 (35.7)	6 (21.4)	0.17
>3 hours/day, n(%)	9 (32.1)	6 (21.4)	
History of corticosteroid use, n (%)	5 (20.0)	5 (22.7)	0.82
Current smoker, n (%)	6 (21.4)	2 (7.1)	0.13
Little to no exercise, n (%)	18 (64.3)	13 (46.4)	0.18
BMI >25 kg/m^2^, n (%)	18 (64.3)	17 (60.7)	0.60

Abbreviations: eGFR, estimated glomerular filtration rate; MDRD, modified diet in renal disease formula; NRTIs, nucleoside reverse transcriptase inhibitor; BMI, body mass index.

Mean (±SD) unless otherwise stated, ^†^median (IQR).

P –values are between group differences.

**Table 2:** Vitamin D-related biochemical and lifestyle characteristics.

	Tenofovir	NRTI	P-value
Vitamin D deficient, n (%)	14 (52)	13 (48)	0.759
Vitamin D insufficient, n (%)	5 (18)	10 (37)	
Optimal vitamin D, n (%)	8 (30)	4 (15)	
High PTH, n (%)	7 (25)	8 (30)	0.70
Osteoporosis or osteopaenia, n(%)	11 (39)	5 (19)	0.09
Meeting RNI for calcium, n (%)	7 (26)	6 (21)	0.69
Estimated protein intake, g/kg/d^†^	1.11 (0.93–1.23)	1.14 (1.01–1.45)	0.30
Estimated calcium intake, mg/d^†^	489 (318–648)	419 (234–746)	0.51
Alkaline phosphatase, u/L	93 (±27)	79 (±25)	**0.04**
Serum phosphate, mmol/L	0.95 (±0.18)	0.97 (±0.13)	0.66
Serum adjusted calcium, mmol/L	2.34 (±0.08)	2.34 (±0.08)	0.94
Serum 25(OH)D concentration, nmol/L	53 (±30)	49 (±23)	0.59
Serum 1,25(OH)_2_D concentration, pg/mL^†^	44 (36–50)	38 (32–42)	0.08
Serum PTH concentration, pmol/L	5.9 (±2.4)	5.9 (±2.5)	0.98
Bone specific ALP, u/L	35 (±13)	30 (±10)	0.13
NTX, BCE nmol/mmol Creatinine^†^	38 (30–45)	34 (26–41)	0.35
FGF-23, RU/ml^†^	21 (15–37)	25 (20–33)	0.48
Urinary calcium, mmol/24 h^†^	3.01 (2.15–3.87)	4.58 (3.85–6.18)	**<0.0001**
Fractional excretion of calcium, %^†^	0.63 (0.44–1.01)	1.17 (0.96–1.38)	**0.004**
Urinary phosphate, mmol/24 h^†^	25.44 (21.97–37.77)	28.25 (21.35–38.30)	0.60

Abbreviations: PTH, parathyroid hormone; RNI, reference nutrient intake; NTX, type 1 collage N-terminal telopeptide Xlinks; FGF-23, fibroblast growth factor 23.

Vitamin D deficient (<50 nmol/L), Vitamin D insufficient (50–75 nmol/L), Optimal vitamin D (>75 nmol/L).

Mean (±SD) unless otherwise stated, ^†^Median (IQR).

Having compared PTH and other parameters between the two groups of antiretroviral regimes, we then examined correlates of PTH in all study participants. Factors significantly associated with higher PTH levels were lower 25-hydroxyvitamin D, female sex, non-white ethnicity, lower urinary excretion of calcium, lower urinary excretion of phosphate, lower dietary intake of calcium, percentage lean body mass, percentage total body fat, reported sun exposure, serum calcium, and 1,25(OH)_2_D:25(OH)D ratio (see Table S2). Reported sun exposure (being outdoors in daylight <15 minutes, 15–29 minutes, 30–59 minutes, 1–2 hours, 3–4 hours or >4 hours daily) was a stronger predictor of 25(OH)D (B coefficient 8.8; p<0.0001) and PTH (B coefficient −0.5; p = 0.013) than was season alone (B coefficient 16.9; p = 0.02 and B coefficient −0.8; p = 0.24 respectively).

Multiple linear regression analysis of determinants of PTH revealed significant interactions between ethnicity, sex and tenofovir use, after adjusting for age (see Table 3). Higher PTH levels were significantly associated with older age and non-white ethnicity. For the interacting effects found, males on TDF had higher PTH levels than males on other NRTIs, and non-white males on TDF had higher PTH levels than both non-white and white males on other NRTIs, and white males on TDF. TDF use alone was not significantly associated with PTH levels, nor was sex.

**Table 3:** Multivariate linear regression analysis of serum PTH levels.

Variable	Coefficient B (95% CI)	SE	P-value
ART	4.06 (0.86 to 7.26)	1.60	0.69
Sex	1.13 (−1.08 to 3.35)	1.10	0.30
Ethnicity	3.54 (1.90 to 5.17)	0.81	0.003
Age	0.08 (0.03 to 0.13)	0.03	0.004
ART*Sex	−3.81 (−6.81 to −0.81)	1.49	0.014**
ART*Ethnicity	−3.29 (−5.75 to −0.84)	1.22	0.01**

** p-value for interaction effect.

R^2^ = 0.515 (Adjusted R^2^ = 0.452).

Abbreviations: ART, antiretroviral therapy.

**Figure 1:**
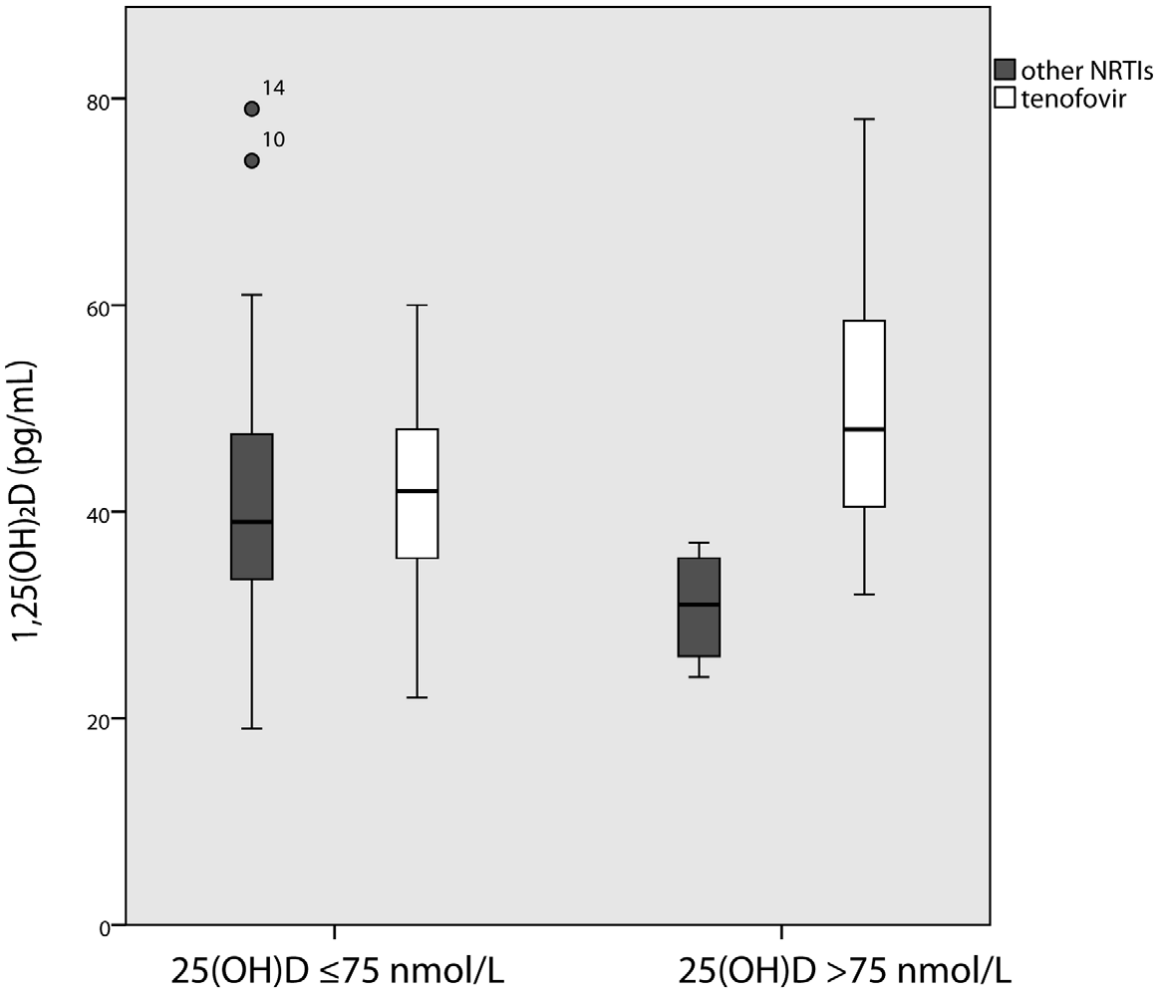
1,25(OH)_2_ vitamin D differences according to 25(OH)vitamin D and ART regime.

As vitamin D (25(OH)D) status has shown to have different effects on PTH and calcium metabolism in patients on tenofovir in previous studies, we then explored these differences in our cohort. In patients with optimal 25(OH)D levels, those on TDF had higher serum 1,25(OH)_2_D concentration (median 53 pg/mL, IQR 41–61 versus 31, 26–36; p = 0.008, figure 1), fractional excretion of phosphate (median 29.8%, IQR 20.3–31.9 versus 15.6, 7.8–16.6; p = 0.05) and lower serum phosphate (median 0.77 mmol/L, IQR 0.71–1.02 versus 1.06, 1.02–1.11; p = 0. 026) than those on other NRTIs, however, there was no difference in fractional excretion of calcium (median 1.06%, IQR 0.60–1.39 versus 1.16%, 1.05–1.87; p = 0.61) or FGF-23 levels. These differences were not seen in those patients who were vitamin D deficient or insufficient, however, in those with vitamin D insufficiency (≤75 nmol/L), those on TDF had significantly lower fractional excretion of calcium (0.56%, IQR 0.42–0.73 versus 1.17%, 0.81–1.38; p = 0.002).

## Discussion

We have carried out a detailed and comprehensive study of bone, calcium and vitamin D metabolism in relation to TDF, which has added to our understanding of the effect on TDF on calcium and vitamin D metabolism. We found that those on TDF had significantly reduced urinary calcium excretion and that vitamin D status modified the effect of TDF on markers of calcium and phosphate metabolism. Patients on TDF had higher levels of PTH when corrected for other factors, but the effect was only seen in men who were not of white ethnicity. This is partially consistent with other observational studies where TDF has been associated with increased PTH levels [9]–[11], [17].

The physiological response to deficiency of either vitamin D or calcium is to stimulate PTH secretion. This in turn stimulates the kidneys to produce 1,25(OH)_2_D, enhances tubular reabsorption of calcium (thereby decreasing urinary calcium excretion) and also activates osteoblasts, leading to an increase in bone alkaline phosphatase activity [18].

In our study, patients on TDF with optimal 25(OH)D levels (>75 nmol/L), had higher 1,25(OH)_2_D levels, a higher fractional excretion of phosphate and lower serum phosphate levels than on other NRTIs. These differences were not seen in the presence of vitamin D deficiency. This suggests that the increased 1 α-hydroxylation rates and tubular phosphate losses, which drive calcium preservation and possibly altered bone metabolism commonly seen in patients on TDF, is dependent on vitamin D status. Previous studies have had conflicting findings, although it is difficult to directly compare these due to the different methodologies and laboratory parameters used. Our study supports the findings of a prospective Swiss cohort of mainly white men which found that patients on TDF had higher 1 α-hydroxylation rates compared to those on other ART only in those patients with optimal 25(OH)D levels [19]. Others [10], [11] have reported that TDF was associated with increased PTH levels in those who were vitamin D deficient and no differences in PTH levels in those who were vitamin D sufficient. There are several potential explanations for the alteration we have seen. Several studies have reported increased renal losses of phosphate in those on TDF. This has been speculated to be because of either tubular damage due to the accumulation of TDF within tubular epithelial cells [20] or a resetting of the renal phosphate threshold [21]. Regardless of the reason behind increased hyperphosphaturia, this may feedback to the complex PTH/1,25(OH)_2_D/calcium pathway. The normal physiological response to vitamin D deficiency is an increase in PTH stimulation and a subsequent reduction in renal phosphate reabsorption. The urinary phosphate losses seen in those on TDF therefore, may be masked by vitamin D deficiency and only become apparent in the setting of optimal vitamin D status when they are evidently ‘unphysiological’. Furthermore, several case reports of tenofovir-associated Fanconi syndrome have reported that the majority of these cases had normal 25(OH)D and PTH levels [22], [23], further supporting this theory. Due to the small numbers in our study, we were unable to further explore this, and as there have also been conflicting findings in the literature, a larger longitudinal study would help to further explain the effects of vitamin D status on calcium and phosphate metabolism in those on TDF.

The emphasis of this study was on the detailed exploration of the metabolic consequences of TDF. In particular, several variables have not been well examined in adults on antiretroviral therapy, including FGF-23, personal UV exposure and calcium metabolism.

FGF-23 is the most extensively studied phosphatonin which is involved in phosphate regulation [24], however the effect that TDF use may have on its stimulation or catabolism has not been reported. Our study demonstrated that FGF-23 levels did not differ between TDF and non-TDF containing ART, however two patients receiving TDF and NVP were found to have extremely high FGF-23 levels (>300 RU/mL). Additionally, we did not see a difference in FGF-23 levels in those with optimal vitamin D status and increased 1,25(OH)_2_D. Bech et al [21] recently reported that when comparing two groups of patients on TDF with and without hypophosphataemia, no differences were seen in FGF-23 levels between the two groups, questioning the role of FGF-23 in altered phosphate metabolism sometimes seen in HIV-infected patients on TDF. We used a C-terminal assay to measure FGF-23, which was also used by Bech et al, that measures both the intact and biologically inactive portions of FGF-23. Burnett et al [25] compared the differences between the C-terminal and intact assays. The intact FGF-23 assay showed the effects of the associations between FGF-23 and serum phosphate, fractional excretion of phosphate and 1,25(OH)_2_D more clearly than the C-terminal assay. Therefore evaluating intact FGF-23 in patients on TDF would be worthwhile in future studies. Abnormally elevated FGF-23 levels are seen in several pathological conditions and are associated with hypophosphataemia, low 1,25(OH)_2_D, urinary phosphate wasting and osteomalacia. One study in Gambian children with rickets found extremely high FGF-23 in these patients along with high 1,25(OH)_2_D and low dietary calcium [26]. The two women in our study with high FGF-23 also had high 1,25(OH)_2_D levels and low dietary calcium intakes, however they did not have phosphaturia and had normal bone mineral density which is unexpected and appears to signify an abnormality in phosphate metabolism in these individuals.

In this study, reported time outdoors as a surrogate of personal UV exposure was a stronger determinant of both 25(OH)D and PTH than season alone. Photophobia, photosensitivity, immobility, depression and sun avoidance due to the perceived increased risk of skin cancer may be potential factors altering sun exposure practices in people living with HIV. We are the first to describe a lower 24 hour urinary calcium excretion and fractional excretion of calcium in patients on TDF with eight patients on TDF with urinary calcium excretion lower than the reference range. No differences in urinary calcium excretion were found in a longitudinal study comparing patients starting on TDF versus ABC [10]. These patients were only followed until week 48, and our cohort comprised of patients who had been on ART for greater than 52 weeks, therefore it is possible that the cumulative effect of TDF use may decrease calcium excretion. Higher 1,25(OH)_2_D levels in patients on TDF were found in this study and in others [19], therefore the finding of reduced calcium excretion would be in keeping with the physiological response to an increased 1,25(OH)_2_D level, but it appears to be unrelated to the increased fractional excretion of phosphate also found in those on TDF. As we used LC-MS/MS to determine 25(OH)D, which can differentiate between 25(OH)D and other metabolites, including 24,25(OH)_2_D, which may be altered in those on ART, we can rule out possible cross-reactivity with 24,25(OH)_2_D in our 25(OH)D measurement. The clinical significance of this finding cannot be determined from a cross-sectional analysis.

Many of our participants had very low intakes of dietary calcium as only 24% met the RNI for calcium (700 mg/day). In the general population, the mean intake of dietary calcium is well above the RNI for both men and women [27]. There are often ethnic differences in calcium intake, especially historical intake, and it has been reported that some people of African origin have higher PTH levels which may be due to their low dietary calcium intake [28]. In our study, those of African origin had significantly lower intakes of dietary calcium (data not shown). Dietary calcium deficiency has been associated with increased parathyroid hormone secretion especially in the presence of 25(OH)D deficiency [29]. However, many studies evaluating PTH levels in patients with HIV infection have not examined dietary influences on PTH levels. Our study also found that in simple linear regression, lower dietary calcium intakes were associated with higher PTH levels. Therefore the interplay between dietary calcium intake and ethnicity should be considered, especially in ethnically diverse cohorts.

Limitations in this study include the small numbers in each group of patients, and the absence of white women which limited the ability to detect differences in the ethnicity subanalyses. In addition, the length of time taking NRTIs, the NNRTI used and season of biochemical measurement was significantly different between the groups. This was primarily due to the increase in prescribing of TDF after its approval by the European Medicines Agency in 2002. However, these variables were not predictors of PTH in either the simple linear or multivariate analyses.

Larger, multi-ethnic studies are needed to confirm our findings about the interaction between TDF use and non-white males. Additionally, future studies should examine prospectively the effect of vitamin D status and vitamin D supplementation on calcium and phosphate metabolism, and the contributing effect of different antiretroviral regimes, to try and clarify these relationships.

## Conclusions

The effect of TDF on PTH levels seems to be dependent on sex and ethnicity. This study was not able to demonstrate a link between hyperparathyroidism and altered bone metabolism nor renal tubular dysfunction frequently seen in patients on TDF. Vitamin D deficiency appeared to be ‘overriding’ the effect of TDF on 1 α-hydroxylation of vitamin D. Urinary calcium excretion was significantly lower in all patients on TDF. Future studies should confirm this as there have been inconsistent findings from other studies.

## Supporting Information

Table S1 **Vitamin D-related biochemical and lifestyle characteristics in non-white males.**
(DOC)

Table S2 **Univariate associations with PTH concentrations.**
(DOC)
