# Supplementary material for: The Effect of Tenofovir on Vitamin D Metabolism in HIV-Infected Adults Is Dependent on Sex and Ethnicity
Source: PLoS One. 2012 Sep 12;7(9):e44845. doi: 10.1371/journal.pone.0044845 (PMC3440360; doi:10.1371/journal.pone.0044845)
Supplement: Table S1 — Vitamin D-related biochemical and lifestyle characteristics in non-white males. (DOC) [file pone.0044845.s001.doc]

**Table S**1. Vitamin D-related biochemical and lifestyle characteristics in non-white males

|  | **Tenofovir (n=5)** | **NRTI (n=4)** | **P-value** |
| --- | --- | --- | --- |
| Vitamin D deficient, n (%) | 4 (80) | 3 (75) | 1.0 |
| Vitamin D insufficient, n (%) | 0 (0) | 1 (25) |  |
| Optimal vitamin D, n (%) | 1(20) | 0 (0) |  |
| High PTH, n (%) | 4 (80) | 0 (0) | 0.048 |
| Osteoporosis or osteopaenia, n(%) | 1 (20) | 0 (0) | 1.0 |
| Meeting RNI for calcium, n (%) | 4 (80) | 2 (50) | 0.52 |
| Estimated protein intake, g/kg/d* | 1.18 (1.11-1.22) | 1.47 (1.47-1.47) | 0.38 |
| Estimated calcium intake, mg/d* | 527 (259-551) | 546 (261-929) | 0.56 |
| Alkaline phosphatase, u/L | 68 (56-70) | 71 (62-82) | 0.73 |
| Serum phosphate, mmol/L | 0.94 (0.84-1.01) | 1.00 (0.97-1.04) | 0.19 |
| Serum adjusted calcium, mmol/L | 2.29 (2.24-2.36) | 2.29 (2.28-2.32) | 1.00 |
| Serum 25(OH)D concentration, nmol/L | 31 (26-41) | 36 (23-54) | 1.00 |
| Serum 1,25(OH)_2_D concentration, pg/mLᶧ | 36 (35-39) | 45 (36-62) | 0.56 |
| Serum PTH concentration, pmol/L | 8.5 (7.6-10.2) | 4.6 (4.0-5.9) | 0.03 |
| Bone specific ALP, u/L | 35 (24-37) | 28 (25-32) | 0.56 |
| NTX, BCE nmol/mmol Creatinine* | 26 (26-30) | 32 (31-48) | 0.14 |
| FGF-23, RU/mlᶧ | 15 (14-19) | 24 (21-31) | 0.06 |
| Urinary calcium, mmol/24 h* | 2.28 (1.91-3.04) | 7.15 (6.18-8.11) | 0.10 |
| Fractional excretion of calcium, %* | 0.56 (0.44-0.68) | 1.30 (1.21-1.38) | 0.10 |
| Urinary phosphate, mmol/24 h* | 25.67 (25.44-27.96) | 45.09 (39.97-50.20) | 0.10 |

Abbreviations: PTH, parathyroid hormone; RNI, reference nutrient intake; NTX, type 1 collage N-terminal telopeptide Xlinks; FGF-23, fibroblast growth factor 23

Vitamin D deficient (<50 nmol/L), Vitamin D insufficient (50-75 nmol/L), Optimal vitamin D (>75 nmol/L)

Mean (±SD) unless otherwise stated, *Median (IQR)
