# Supplementary material for: The Effect of Tenofovir on Vitamin D Metabolism in HIV-Infected Adults Is Dependent on Sex and Ethnicity
Source: PLoS One. 2012 Sep 12;7(9):e44845. doi: 10.1371/journal.pone.0044845 (PMC3440360; doi:10.1371/journal.pone.0044845)
Supplement: Table S2 — Univariate associations with PTH concentrations. (DOC) [file pone.0044845.s002.doc]

**Table S**2. Univariate associations with PTH concentrations

| Variable | B (95% confidence interval) | P-value |
| --- | --- | --- |
| Serum alkaline phosphatase | 0.02 | 0.06 |
| Serum bone specific alkaline phosphatase | 0.06 | 0.05 |
| Serum adjusted calcium | -9.1 | 0.03* |
| Serum 25(OH)D | -0.04 | 0.001* |
| 1,25(OH)_2_D:25(OH)D | 1.0 | 0.02* |
| 24-hour urinary calcium excretion** | -2.2 | 0.04* |
| Fractional excretion of calcium | -1.2 | 0.07 |
| 24-hour urinary phosphate excretion** | -5.8 | 0.009* |
| Fractional excretion of phosphate | -0.05 | 0.37 |
| % lean body mass (muscle) | -0.08 | 0.007* |
| % total body fat | 0.09 | 0.002* |
| % android fat | 0.08 | 0.02* |
| Age | 0.05 | 0.14 |
| Dietary calcium intake (including supplements) | -0.002 | 0.001* |
| Sex (male vs female) | 2.7 | <0.0001* |
| Ethnicity (nonwhite vs white) | -2.7 | <0.0001* |
| Outdoors >30 minutes/day in past month | -2.7 | <0.0001* |
| Season (non-summer months vs summer) | -0.77 | 0.24 |
| TDF vs. Other NRTIs | -0.02 | 0.98 |
| Nevirapine vs efavirenz | -0.30 | 0.67 |

*p<0.05

**log-transformed

Abbreviations: TDF – tenofovir disoproxil fumarate; NRTI – nucleoside reverse transcriptase inhibitor
